# Carvacrol Induces *Candida albicans* Apoptosis Associated With Ca^2+^/Calcineurin Pathway

**DOI:** 10.3389/fcimb.2020.00192

**Published:** 2020-04-30

**Authors:** Chao Niu, Chenglu Wang, Yijia Yang, Ruiyao Chen, Jian Zhang, Haiyan Chen, Yingzhi Zhuge, Jingqi Li, Jianhua Cheng, Ke Xu, Maoping Chu, Chunhua Ren, Chunxiang Zhang, Chang Jia

**Affiliations:** ^1^Pediatric Research Institute, The Second Affiliated Hospital and Yuying Children's Hospital of Wenzhou Medical University, Wenzhou, China; ^2^The Second Clinical Medical College of Wenzhou Medical University, Wenzhou, China; ^3^Children's Heart Center, Institute of Cardiovascular Development and Translational Medicine, The Second Affiliated Hospital and Yuying Children's Hospital of Wenzhou Medical University, Wenzhou, China; ^4^Department of Neurology, The First Affiliated Hospital of Wenzhou Medical University, Wenzhou, China; ^5^The Institute of Life Sciences, Wenzhou University, Wenzhou, China; ^6^Tianjin Key Laboratory of Radiation Medicine and Molecular Nuclear Medicine, Institute of Radiation Medicine, Chinese Academy of Medical Sciences and Peking Union Medical College, Tianjin, China

**Keywords:** *C. albicans*, carvacrol, apoptosis, calcineurin, immunomodulation

## Abstract

As the prevalence of systemic fungal infections caused by *Candida albicans* gradually increases, it is necessary to explore potential and effective antifungals. Carvacrol is reported to be lethally toxic to *C. albicans*, involving several potential mechanisms. However, the form and specific mechanism of cell death caused by this compound has not been delineated. In this study, we found that carvacrol could significantly decrease *C. albicans* survival rates, consistent with previous researches. Further examination proved that carvacrol treatment caused cell membrane permeability and depolarization. To elucidate the association between cell death and apoptosis, DNA fragmentation and metacaspase activation were determined; as expected, these two apoptosis-related markers were clearly observed. Moreover, total and mitochondrial reactive oxygen species (ROS) levels were elevated, and both mitochondrial transmembrane potential and morphology were disrupted. Additionally, cytosolic and mitochondrial calcium levels were also increased by carvacrol. Calcineurin inhibition experiments revealed cyclosporine A (CsA) addition notably rescued cell growth and inhibited metacaspase activation, indicating that carvacrol triggered *C. albicans* apoptosis through inducing calcineurin activation. Carvacrol was demonstrated to both have low toxicity and be effective in alleviating systemic infections with *C. albicans*, which might be via its antifungal and immunomodulation activities. This study suggests that carvacrol has excellent potential as a natural protective compound against *C. albicans* infections.

## Introduction

*Candida albicans* can lead to both topical epithelial and fatal invasive infections in immunocompromised patients, contributing to its status as the fourth most common cause of nosocomial blood-stream infections in US hospitals (Klepser, 2006). The increased systemic infections resulting from *C. albicans* underly mortality rates of at least 50% even though there are presently available antifungal therapy (Pfaller and Diekema, 2007). Therapeutic options are currently restricted to the utilization of the three longstanding antifungal classes: azoles, polyenes, and echinocandins (Chaillot et al., 2015). However, these abovementioned drugs have severe side effects, including nephrotoxicity and fungal resistance due to their fungistatic rather than fungicidal effects (Shapiro et al., 2011). Therefore, it is urgent to explore novel and more effective strategies for antifungal therapeutic intervention.

Traditional Chinese medicines provide an interesting reservoir of potentially useful and effective antimicrobial secondary metabolites. Carvacrol, a monoterpene phenol, is major component of essential oil extracts from plants in the family *Lamiaceae* including oregano (*Origanum vulgare* L.) and marjoram (*Origanum marjoram* L.) (Nunes Wolffenbuttel et al., 2015). This phytomolecule in low concentration is considered safe for humans and it is commonly applied as a flavoring agent (Suntres et al., 2015). In addition, this compound has extensively demonstrated pharmacological properties, including antifungal and antibacterial activities, and immunoregulatory potential (Wieten et al., 2010). Previous studies have reported that carvacrol can impede the growth of different morphological forms of *C. albicans*, including yeast, hyphae, and biofilm (Inouye et al., 2009; Lima et al., 2013; Raut et al., 2013). Moreover, the anti-*C. albicans* activity of carvacrol has also been substantiated in the rat vaginal candidiasis model (Chami et al., 2004b) and murine systemic candidiasis model (Manohar et al., 2001). However, the antifungal activity of this compound has not been completely elucidated *in vivo*. Currently reported mechanisms of action for this phytomolecule include disrupting ergosterol biosynthesis and membrane integrity (Ahmad et al., 2011), inducing endoplasmic reticulum (ER) stress and the unfolded protein response (UPR) (Chaillot et al., 2015), and perturbing H^+^ and Ca^2+^ ion homeostasis (Rao et al., 2010). Although all the aforementioned modes of action of carvacrol can kill *C. albicans*, the form and ultimate mechanism of cell death in *C. albicans* caused by carvacrol remains unclear.

In the present study, to expound the types of cell death, various parameters related to apoptosis were assessed in *C. albicans* cells treated with carvacrol. In addition, the antifungal effect and immunoregulation activity of carvacrol were also determined in a murine model of *C. albicans* infection. Our study elucidated that the antifungal efficacy of carvacrol against *C. albicans* was realized through inducing apoptosis. Furthermore, carvacrol exhibited obvious antifungal and immunoregulation properties in the murine systemic candidiasis model.

## Materials and Methods

### Chemicals, Strains, and Cultures

**Figure 1:**
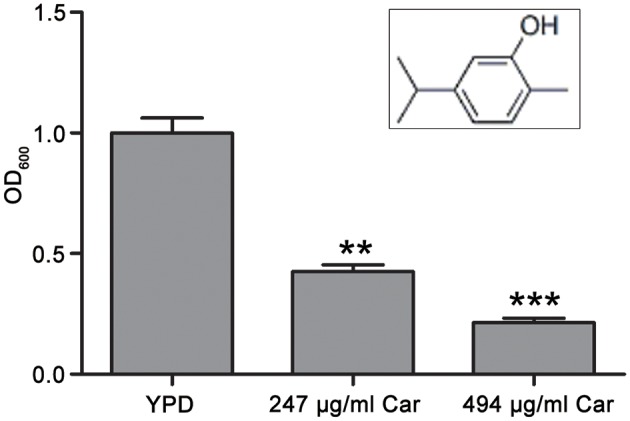
Chemical structure of carvacrol and its effect on *C. albicans* survival. The chemical structure of carvacrol was exhibited, and the effect of carvacrol on *C. albicans* survival was analyzed by determining CFUs. Data were shown as mean ± SD (*n* = 5). Significant difference was designated as **P < 0.01 and ***P < 0.001.

Carvacrol (>99%, CAS: 499-75-2) was obtained from Weikeqi Biotechnology Co., Ltd. (Sichuan, China). Its chemical structure was shown in Figure 1. A stock carvacrol (494 mg/ml) was made by dissolving this compound in dimethyl sulfoxide (DMSO). The three strains of *C. albicans* used in this study were SC5314, ATCC18804, and 07318, and each was cultured at 30°C in yeast extract peptone dextrose (YPD) medium.

### Antifungal Activity Testing

Microdilution methods were used to determine the minimal inhibitory concentration (MIC). Specifically, aliquots of 100 μl of *C. albicans* cell suspensions (1 × 10^5^ cells/ml) with different carvacrol concentrations were transferred into a series of 96-well plates. After a 48-h incubation at 30°C, the MIC of carvacrol against SC5314 strain was examined (Tian et al., 2017; Yun and Lee, 2017). This experiment was conducted in triplicate.

Overnight cultures were diluted in YPD medium to an OD_600_ value of 0.1. After reaching the mid-exponential phase, *C. albicans* cells cultures were subjected to 3-h treatments with 247 and 494 μg/ml carvacrol. The treated cells were then collected, and about 10^3^ cells were coated onto plates of YPD agar. Following a 24-h aerobic incubation at 30°C, the numbers of colony forming units (CFUs) were determined. Percentage survival estimates were detected relative to the untreated control cells, and each experiment was conducted independently in triplicate.

### Cell Membrane Integrity Assay

Analysis of cell membrane integrity was conducted according to a modified version of the method described by Li et al. (2016). Specifically, after *C. albicans* cells were treated with carvacrol, they were collected, and stained for 30 min at 4°C with 10 mg/ml propidium iodide (PI) in darkness. Then, cell membrane integrity was assayed using a FACSCalibur flow cytometer (Becton Dickinson, United States).

### Plasma Membrane Potential Determination

DiBAC_4_(3)(bis-(1,3-dibarbituric acid)-trimethine oxanol) was used as a membrane potential molecular probe to assess changes in plasma membrane potential as previously described (Li et al., 2016). In brief, *C. albicans* cells were treated, harvested, and washed thrice with phosphate-buffered saline (PBS). Next, the *C. albicans* cell suspension was incubated with 20 mg/ml DiBAC_4_(3) at 37°C for 30 min in darkness. Depolarized *C. albicans* cells were examined by flow cytometry, and the percentage of depolarized cells was recorded.

### Metacaspase Activation Assay

The CaspACE FITCVAD-FMK *in situ* marker (Promega, Madison, WI, USA) was utilized to detect metacaspase activation (Tian et al., 2017). Specifically, carvacrol-treated *C. albicans* cells were collected and washed with PBS. Then, cells staining was conducted with CaspACE FITC-VAD-FMK at a 5 mM final concentration at 37°C for 20 min in darkness. Next, the stained cells were again washed with PBS and assayed using flow cytometry.

### DNA Fragmentation Evaluation

Terminal deoxynucleotidyl transferase dUTP nick end labeling (TUNEL) staining was utilized to examine *C. albicans* DNA fragmentation (Jia et al., 2018). Specifically, treated *C. albicans* were harvested, washed with PBS, and fixed for 30 min in 3.6% paraformaldehyde followed by a 2-min permeabilization on ice. Next, the cells were again washed before being stained with an *in situ* cell death detection kit at 37°C for 1 h in darkness. DNA fragmentation was finally assessed by flow cytometry.

### Intracellular ROS Measurement

To measure *C. albicans* reactive oxygen species (ROS) levels, the fluorescent probe 2′, 7′–dichlorofluorescein diacetate (DCFH-DA) was utilized. DCFH-DA can be cleaved by intracellular esterase into the membrane-impermeable agent DCFH, which ROS can then oxidize into its fluorescent derivative, DCF. Thus, the fluorescence intensity of DCF is an indicator of the total ROS levels (Jia et al., 2019). Accordingly, carvacrol-treated *C. albicans* cells were gathered, washed with YPD medium one time, and resuspended in 0.5 ml of YPD medium containing 10 mM DCFH-DA. Following a 30-min incubation in the dark, the fluorescence intensity of *C. albicans* cells was assessed using flow cytometry.

### Mitochondrial Superoxide Anion Determination

MitoSOX Red mitochondrial superoxide indicator was utilized to determine the mitochondrial ROS levels (Zhou et al., 2019). Specifically, carvacrol-treated *C. albicans* cells were collected, washed, stained with MitoSOX Red mitochondrial superoxide indicator at a final concentration of 5 μM, and incubated for 20 min at 37°C. After staining, cells were harvested and assayed using a flow cytometer.

### Mitochondrial Membrane Potential Determination

To examine the mitochondrial transmembrane potential, 5,5′,6,6′-tetrachloro-1,1′,3, 3′-tetraethyl-benzimidazolyl carbocyanine iodide (JC-1) was applied (Jia et al., 2019). Carvacrol-treated *C. albicans* cells were gathered by centrifugation, twice washed with PBS, and then incubated for 20 min with 2.5 μg/ml JC-1 at 37°C in darkness. After the stained cells were washed again with PBS, they were assayed using a FACSCalibur flow cytometer. The mitochondrial membrane potential was then estimated as the ratio of the fluorescence intensity of JC-1 aggregates (FL2) to that of its monomer (FL1).

### Examination of Mitochondrial and Cytosolic Calcium Levels

Rhod-2 AM and Fluo-3 AM were, respectively, used to examine the mitochondrial and cytosolic calcium levels (Tian et al., 2017; Jia et al., 2018). *C. albicans* cells were treated with 247 and 494 μg/ml carvacrol. Next, the carvacrol-treated *C. albicans* cells were centrifuged, collected, washed twice with Hank's balanced salt solution (HBSS), and resuspended in 500 μl of HBSS buffer. To assay mitochondrial calcium levels, the washed *C. albicans* cells were stained with 4 mM Rhod-2 AM for 30 min at 37 °C in the dark. To measure cytosolic calcium levels, cells were stained with 2 mM Fluo-3 AM for 40 min at 30°C in the dark. Next, the stained cells were washed again, resuspended in 600 μl of HBSS, and incubated for another 20 min at 30°C. The fluorescence intensities of Rhod-2 AM and Fluo-3 AM were then immediately detected with a flow cytometer.

### Murine Model of *C. albicans* Infection

Whether carvacrol can clear *C. albicans* infections was determined by infecting female Institute of Cancer Research (ICR) mice (25–30 g) with 100 μl of a 5 × 10^6^ *C. albicans* cells/ml normal saline suspension via tail vein injection. One hour post-infection, 200 μl of normal saline, 16 mg/kg carvacrol, and 32 mg/kg carvacrol were, respectively, administered via oral-gastric (OG) gavage, and this was repeated daily on days 2–5. Each group included six mice. Mice survival was assessed on day 10. Mice were sacrificed on the fourth day after infection in order to assess the fungal burdens in their kidneys, which were then homogenized. Fungal loads were examined by plating dilutions of the resulting homogenate onto YPD agar plates supplemented with 50 μg/ml ampicillin and 100 μg/ml streptomycin followed by a 2-days incubation at 30°C. To evaluate the toxicity of carvacrol, ICR female mice (25–30 g) were administrated with carvacrol at doses of 16 and 32 mg/kg body weight once daily for 5 days by OG gavage with each group comprising six mice, and the survival of mice was monitored over the course of 10 days. SPSS (Version 17.0) was used to conduct data analyses, with the Kaplan–Meier logrank test used to examine the statistical differences between groups, and significant *P*-values noted by asterisks.

### Evaluation of Immunomodulatory Effect of Carvacrol

To examine the effect of carvacrol on RAW264.7 cell viability, RAW264.7 cells (10^5^ cells per well) were incubated at 37°C and 5% CO_2_ in 96-well plates for 24 h to facilitate cell attachment and spreading. Then cells were treated with 0.15625, 0.3125, 0.625, 1.25, or 2.5 μg/ml carvacrol for 24 h. Next, a CCK8 assay was conducted to assess cell viability.

To detect the anti-inflammatory activity of carvacrol, RAW264.7 cells were pretreated with carvacrol for 24 h before being stimulated with 1 μg/ml lipopolysaccharide (LPS) for 4 h. Next, cells were harvested, and their total RNA was extracted. Then, RT-PCR was used to determine the mRNA levels of tumor necrosis factor-α (TNF-α), interleukin-1β (IL-1β), and interleukin-6 (IL-6). Table 1 showed the primers utilized in this study.

**Table 1:** Lists of primers used in this study.

**Primer**	**Sequence (5^**′**^-3^**′**^)**
GAPDH-5RT	AAGAAGGTGGTGAAGCAGG
GAPDH-3RT	GAAGGTGGAAGAGTGGGAGT
TNF-α-5RT	CTTGTTGCCTCCTCTTTTGCTTA
TNF-α-3RT	CTTTATTTCTCTCAATGACCCGTAG
IL-6-5RT	TCACAGAAGGAGTGGCTAAGGACC
IL-6-3RT	ACGCACTAGGTTTGCCGAGTAGAT
IL-1β-5RT	TGTGTTTTCCTCCTTGCCTCTGAT
IL-1β-3RT	TGCTGCCTAATGTCCCCTTGAAT

### Statistical Analysis

In this study, each experiment was independently conducted at least in triplicate. Data were expressed as mean ± standard deviation (SD) value, and were analyzed using SPSS version 17.0. All data generated did not deviate from a normal distribution. Thus, Duncan's Multiple Range tests following one-way analysis of variance were conducted to make comparison among three or more groups, and two-tailed unpaired Student's *t*-tests were used to make comparison between two experimental groups. Statistically significance was indicated by asterisks (*P < 0.05, **P < 0.01, and ***P < 0.001).

## Results

### Effects of Carvacrol on *C. albicans* Survival

The antifungal action of carvacrol against *C. albicans* was investigated. The MIC value of carvacrol against SC5314 strain was 247 μg/ml. To examine the effect of carvacrol on *C. albicans* survival, the CFUs assays were conducted after treatment with this compound for 3 h. The survival percentage was significantly decreased after carvacrol treatment (42.6 ± 2.7 and 21.4 ± 1.8% under 247 and 494 μg/ml carvacrol treatments, respectively) compared with the control (100 ± 6.2%; *P* < 0.05; and *P* < 0.01, respectively; Figure 1), indicating that carvacrol caused *C. albicans* cell death. To better demonstrate the antifungal activity of carvacrol against *C. albicans*, two clinical strains, ATCC18804 and 07318, were treated with carvacrol. Consistent with the above results, similar effects were observed in these clinical strains (data not shown).

### Carvacrol Disturbs *C. albicans* Plasma Membrane

**Figure 2:**
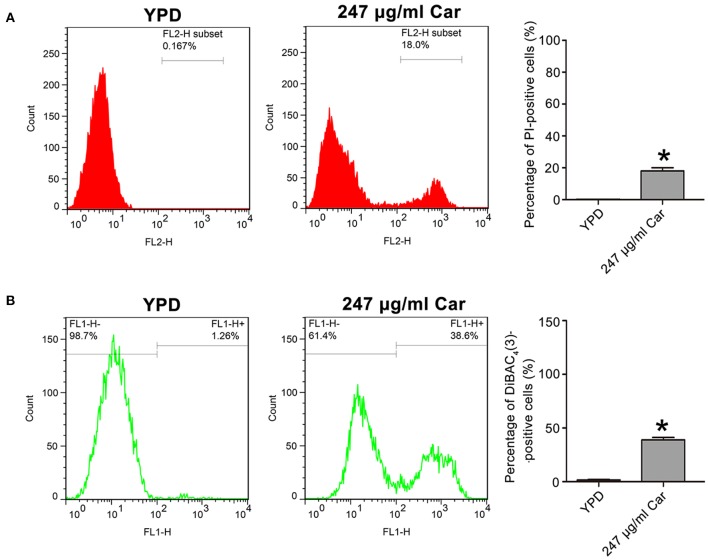
Effects of carvacrol on cell membrane were examined in *C. albicans*. **(A)** Cell membrane integrity was analyzed using PI staining. The histogram showed the percentage of PI-positive cells, and data were expressed as mean ± SD (*n* = 3). *P < 0.05. **(B)** Plasma membrane potential was determined using DiBAC_4_(3) staining. The percentage of DiBAC_4_(3)-positive cells was shown in the histogram, and data were exhibited as mean ± SD (*n* = 3). *P < 0.05.

Previous studies have reported that carvacrol can interfere with ergosterol biosynthesis, which ultimately affects plasma membrane integrity (Chami et al., 2005; Ahmad et al., 2011). To confirm the effect of carvacrol on *C. albicans* cell membranes, cell membrane integrity and potential were, respectively, analyzed using PI and DiBAC_4_(3) staining. As shown in Figure 2A, PI staining analysis revealed a remarkably intense fluorescence signal in 247 μg/ml carvacrol-treated cells (18 ± 2%) compared with control cells (0.15 ± 0.08%, *P* < 0.05; Figure 2A), indicating that carvacrol disrupted *C. albicans* cell membrane integrity. More cells were stained by DiBAC_4_(3) after 247 μg/ml carvacrol treatment (38.97 ± 2.27%) compared with the control cells (1.51 ± 0.5%, *P* < 0.05; Figure 2B). To further assess the results, ATCC18804 and 07318 plasma membranes were also examined. In line with SC5314 observation, carvacrol treatment increased cell membrane permeability and depolarization in the two clinical strains (data not shown). These data together revealed that carvacrol affected both cell membrane integrity and potential in *C. albicans* cells.

### Carvacrol Induces DNA Fragmentation and Metacaspase Activation

**Figure 3:**
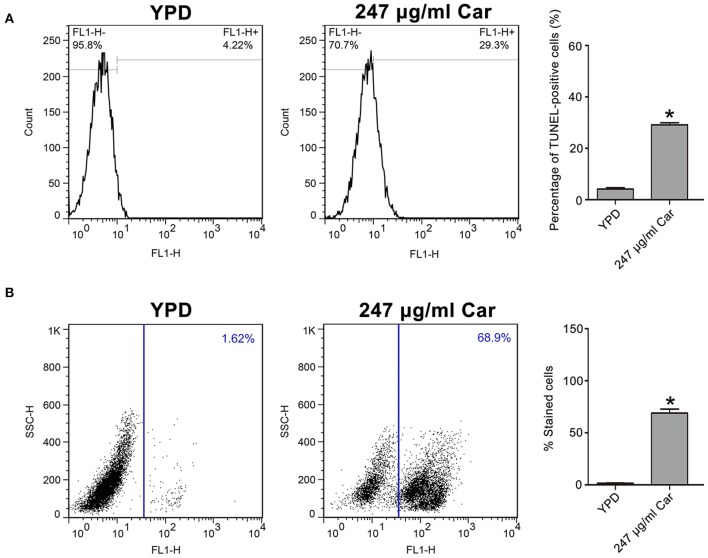
DNA fragmentation and metacaspase activation were analyzed in carvacrol-treated *C. albicans* cells. **(A)** DNA fragmentation was determined using TUNEL staining. The histogram was the quantitative analysis of TUNEL-positive cells, and data were presented as mean ± SD (*n* = 3). *P < 0.05. **(B)** Metacaspase activation was assessed using CaspACE FITC-VAD-FMK *in situ* marker. The percentage of stained cells was shown in the histogram, and data were expressed as mean ± SD (*n* = 3). *P < 0.05.

To further examine the mode of *C. albicans* cell death due to carvacrol, apoptosis-related parameters, including DNA fragmentation and metacaspase activation, were analyzed. As shown in Figure 3A, carvacrol treatment significantly increased the percentage of TUNEL-positive cells (29.1 ± 0.82 vs. 4.12 ± 0.48%, *P* < 0.05; Figure 3A), indicating that DNA fragmentation occurred in carvacrol-treated *C. albicans* cells. Moreover, metacaspase activity was also remarkably increased in *C. albicans* cells following carvacrol treatment (68.97 ± 1 vs. 1.72 ± 0.21%, *P* < 0.05; Figure 3B). These results demonstrate that carvacrol triggered *C. albicans* apoptosis. To confirm this finding, DNA fragmentation and metacaspase activity were also determined in the aforementioned clinical strains. As expected, the percentage of TUNEL-positive cells and metacaspase activity levels were obviously elevated in both clinical strains following carvacrol treatment (data not shown). Collectively, these results indicated that the antifungal action of carvacrol was realized by inducing apoptosis.

### Carvacrol Increases ROS Levels and Affects Mitochondrial Function

**Figure 4:**
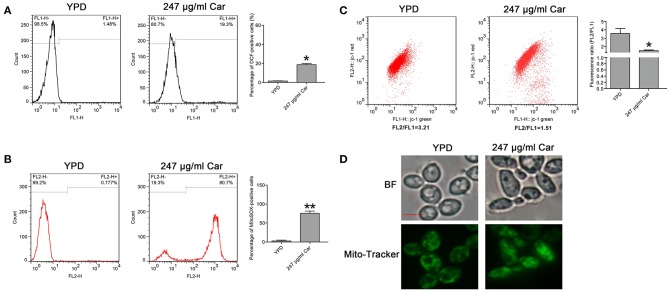
Reactive oxygen species (ROS) level and mitochondrial function were examined in *C. albicans* cells after treatment with carvacrol. **(A)** ROS levels were analyzed using DCFH-DA staining. The histogram showed the percentage of DCF-positive cells, and data were shown as mean ± SD (*n* = 3). *P < 0.05. **(B)** Mitochondrial ROS levels were determined by flow cytometry using a MitoSOX Red mitochondrial superoxide indicator. The histogram showed the percentage of MitoSOX-positive cells, and data were expressed as mean ± SD (*n* = 3). **P < 0.01. **(C)** The mitochondrial membrane potential was assessed using JC-1 staining. The histogram was the quantitative analysis of fluorescence ratio (FL2/FL1), and data was shown as mean ± SD (*n* = 3). *P < 0.01. **(D)** Mitochondrial morphology was observed using Mito-Tracker Green. BF, Bright Field. Bar, 5 μm.

As is known, ROS play a key role in the induction and regulation of apoptotic processes. To examine the effect of carvacrol on ROS levels, the ROS indicator DCFDH-DA was utilized. Carvacrol addition significantly elevated ROS levels (18.67 ± 1.27%) compared with the control (1.55 ± 0.15%, *P* < 0.05; Figure 4A). Mitochondria are a major source of ROS production. Therefore, mitochondrial ROS levels were analyzed using MitoSOX Red, a mitochondrial superoxide indicator. Carvacrol treatment remarkably increased the ROS levels of mitochondria (75.68 ± 5.75% and vs. 2.74 ± 2.15%, *P* < 0.01; Figure 4B). Mitochondrial ROS production is associated with mitochondrial function. Thus, mitochondrial transmembrane potential was evaluated. As expected, mitochondria membrane potential was significantly decreased after treatment with carvacrol (1.52 ± 0.1 and vs. 3.56 ± 0.62%, *P* < 0.05; Figure 4C). Moreover, mitochondrial morphology was remarkably affected by carvacrol (Figure 4D). In addition, we also validated these results in the two clinical strains examined, and similar effects were observed in the carvacrol-treated clinical strains (data not shown). Together, these results indicate that carvacrol treatment disturbed mitochondrial functions.

### Carvacrol Elevates Mitochondrial and Cytosolic Calcium Levels

**Figure 5:**
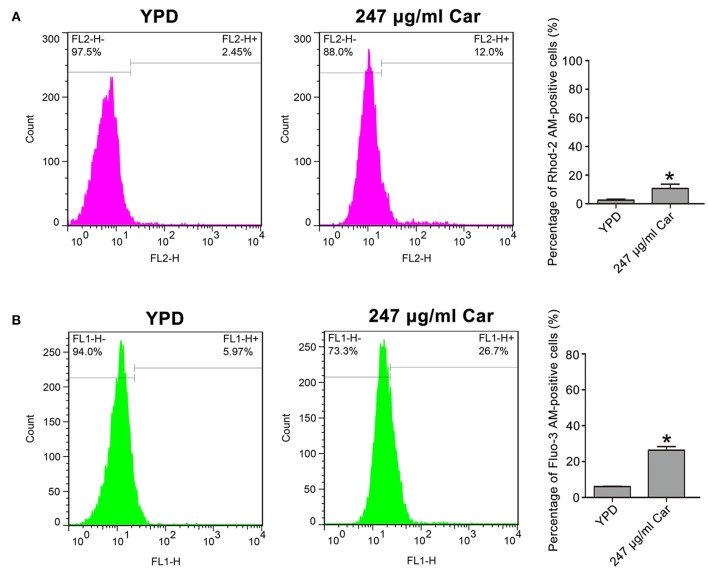
Mitochondrial and cytosolic calcium levels were determined in the treated *C. albicans*. **(A)** Calcium in the mitochondria was examined using Rhod-2 AM. The percentage of Rhod-2 AM-positive cells were shown in the histogram, and the data were presented as mean ± SD (*n* = 3). *P < 0.05. **(B)** Calcium in the cytosol was analyzed via Fluo-3 AM staining. The histogram showed the percentage of Fluo-3 AM-positive cells, and data were exhibited as mean ± SD (*n* = 3). *P < 0.05.

Calcium is known to be essential in apoptotic processes (Pinton et al., 2008). Thus, Rhod-2 AM and Fluo-3 AM were, respectively, utilized to measure mitochondrial and cytosolic and calcium levels. In contrast to the control (2.57 ± 0.56%), there were significantly more Rhod-2 AM-positive carvacrol-treated *C. albicans* cells (10.68 ± 3%, *P* < 0.05; Figure 5B). Moreover, the percentage of Fluo-3 AM-positive cells was notably increased in carvacrol-treated cells (26.4 ± 1.87) compared with control cells (6.04 ± 0.22%, *P* < 0.05; Figure 5A). The two clinical strains responded similarly to SC5314 in terms of calcium changes following carvacrol treatment (data not shown). Thus, carvacrol treatment led to accumulation of both mitochondrial and cytosolic calcium levels in *C. albicans* cells.

### Carvacrol-Induced Apoptosis Is Associated With Ca^2+^/Calcineurin Pathway

**Figure 6:**
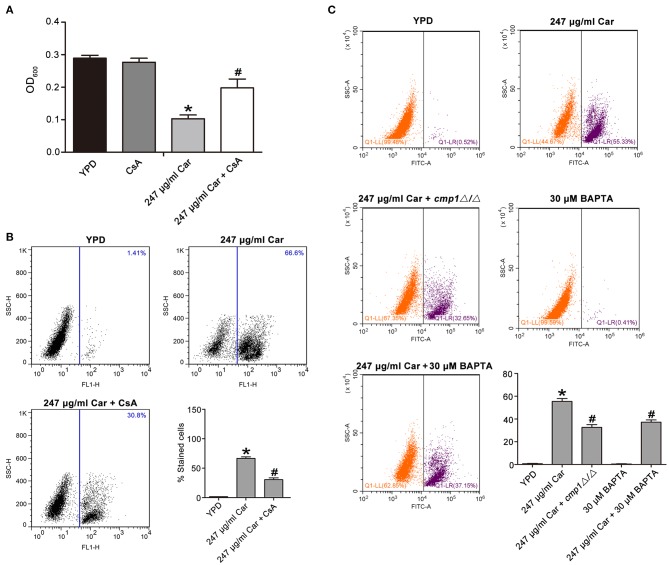
Ca^2+^/calcineruin pathway was involved in carvacrol-induced *C. albicans* apoptosis. **(A)** Effects of CsA on carvacrol-treated *C. albicans* growth were analyzed, and the data was shown as mean ± SD (*n* = 5). **(B)** Metacaspase activation was examined upon pretreated with CsA for 2 h. The histogram showed the percentage of stained cells, and the data were exhibited as mean ± SD (*n* = 3). **(C)** The calcineurin mutant *cmp1*Δ*/*Δ, and calcium-scavenger BAPTA, were used to observe metacaspase activity. The histogram showed the percentage of stained cells, and the data were expressed as mean ± SD (*n* = 3). *P < 0.05 vs. the YPD group; #*P* < 0.05 vs. 247 μg/ml Car group.

Previous studies have reported that calcium can induce calcineurin activation, which induces calcineurin-mediated BAD dephosphorylation and, in turn, activation of the caspase-3 apoptotic cascade, ultimately thereby inducing apoptosis (Wang et al., 1999; Saito et al., 2000; Springer et al., 2000). Another study has also demonstrated that Ca^2+^ and its downstream calcineurin/Crz1p/*CaMCA1* pathway are involved in H_2_O_2_-induced *C. albicans* apoptosis (Lu et al., 2010). Our above findings have demonstrated that calcium levels were increased after carvacrol treatment. Therefore, we anticipated that calcineurin might be involved in carvacrol-induced *C. albicans* apoptosis. To confirm this assumption, a calcineurin inhibitor, cyclosporine A (CsA), was applied to pretreat *C. albicans* for 2 h. Supplementation with CsA significantly rescued *C. albicans* growth (0.147 ± 0.006 vs. 0.088 ± 0.001, *P* < 0.05; Figure 6A), and notably inhibited metacaspase activation (30.45 ± 3.1 vs. 58.4 ± 4.26%, *P* < 0.05; Figure 6B), indicating that carvacrol predisposed *C. albicans* cells to apoptosis via calcineurin activation. To further substantiate that carvacrol induced *C. albicans* apoptosis via Ca^2+^/calcineurin pathway, the calcineurin mutant *cmp1*Δ*/*Δ, and calcium-scavenger BAPTA were used to observe metacaspase activity. As expected, BAPTA addition and *CMP1* deletion significantly decreased carvacrol-mediated metacaspase activity (32.55 ± 2.5% for carvacrol treament plus *CMP1* deletion and 37.15 ± 2% for carvacrol treament plus BAPTA addition vs. 55.44 ± 2.5% for carvacrol treatment only, *P* < 0.05; Figure 6C).

### Carvacrol Mitigates Systemic *C. albicans* Infection in a Murine Model

**Figure 7:**
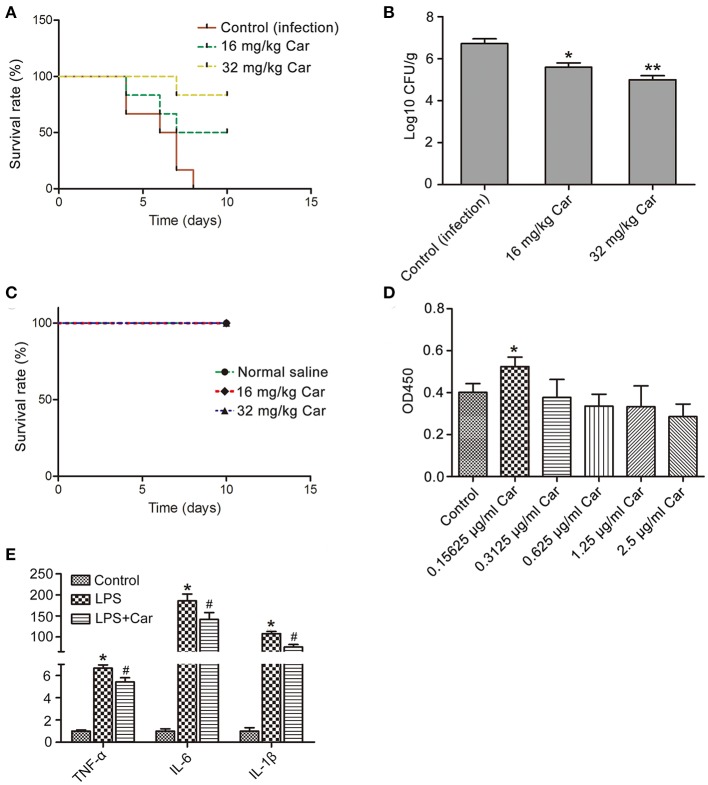
Effects of carvacrol on systemic candidiasis and macrophage were analyzed. **(A)** Survival rate of infected mice was evaluated after treatment with carvacrol. **(B)** The fungal burdens in the kidneys were examined by plating dilutions onto YPD agar plates supplemented with streptomycin and ampicillin. *P < 0.05 and **P < 0.01. **(C)** The toxicity of carvacrol on non-infected mice was observed for 10 days. **(D)** Effect of carvacrol on RAW264.7 macrophage viability was examined after treatment for 24 h, and data were shown as mean ± SD (*n* = 6). *P < 0.05. **(E)** The expression of pro-inflammatory cytokines, including TNF-α, IL-6, and IL-1β, was determined in LPS-stimulated macrophages with or without carvacrol addition. Data were expressed as mean ± SD (*n* = 3).*P < 0.05 vs. the control, and #*P* < 0.05 vs. LPS group.

To assess the effect of carvacrol on *in vivo C. albicans* virulence, this compound was administrated in a mouse model of systemic candidiasis via oral-gastric (OG) gavage. Mice in the infection group died within 10 days, and 50 and 83.3% of mice treated with 16 and 32 mg/kg carvacrol, respectively, survived the entire experiment (^*^P < 0.05 and ^**^P < 0.01) (Figure 7A), indicating that carvacrol could be used to treat *C. albicans* infection. Evaluation of fungal burdens showed that carvacrol administration obviously decreased CFUs in kidney samples from the carvacrol-treated group compared with the control (infection) group (Figure 7B). All uninfected mice survived the entire experiment after administration with carvacrol alone (Figure 7C), indicating that this phytomolecule was non-toxic at doses of 32 mg/kg or less.

Previous studies have reported that carvacrol can modulate the inflammatory response (Du et al., 2016). To elucidate whether carvacrol eliminates *C. albicans* through regulating immunity in addition to its direct antifungal efficacy, we will first find a carvacrol concentration which was effective to promote macrophage viability and proliferation through observing its effects on RAW264.7 macrophage. A low concentration of carvacrol significantly promoted cell viability and proliferation (Figure 7D). Moreover, addition of carvacrol at a low concentration notably down regulated LPS-induced mRNA transcript levels of pro-inflammatory cytokines, including TNF-α, IL-6, and IL-1β (Figure 7E). Taken together, these results indicated that carvacrol diminished *C. albicans* infection through both antifungal and immunomodulation activities.

## Discussion

*C. albicans* is a major opportunistic human pathogen that can cause lethal systemic infections in patients with low immunity. There are limited antifungal agents available for clinical therapy of fungal infections, so the identification of potential new antifungal agents is urgent (Carmona-Gutierrez et al., 2010). Carvacrol has been reported to be able to control the growth of many fungi (Chami et al., 2004b; Ahmad et al., 2011; Numpaque et al., 2011; Lima et al., 2013; Abbaszadeh et al., 2014; Šimovi et al., 2014; Nóbrega et al., 2016), including *C. albicans* (Lima et al., 2013; Chaillot et al., 2015). The involved main mechanism of its action is disruption of endoplasmic reticulum, which eventually leads to the disturbance of many aspects of membrane biology, including permeability and membrane lipids such as ergosterol (Kimura et al., 2006; Ahmad et al., 2011; Chaillot et al., 2015). Although the mechanism of its action has been delineated to some extent, some aspects have remained unclear, such as the form and specific mechanism of cell death in *C. albicans* caused by carvacrol.

In this study, we found that carvacrol could trigger *C. albicans* apoptosis as well as cause membrane disruption. Further examination demonstrated that carvacrol treatment induced ROS production and mitochondrial dysfunction. However, total and mitochondria- specific ROS scavengers, *N*-acetylcysteine (NAC) and mitoTEMPO, failed to rescue the growth inhibition of *C. albicans* caused by carvacrol (data not shown), suggesting that carvacrol treatment led to *C. albicans* apoptosis independent of ROS production. Calcium is also known to participate in the apoptotic process (Pinton et al., 2008). Our data revealed that calcium was accumulated in both the cytosol and mitochondria after treatment with carvacrol, indicating that calcium dysfunction may be involved in carvacrol-induced *C. albicans* apoptosis. A previous study reported that external stresses can activate both the high and low affinity Ca^2+^ influx systems of the plasma membrane, resulting in a rapid influx of Ca^2+^, which then binds to calmodulin and subsequently calcineurin, leading to calcineurin activation, and cell survival (Cruz et al., 2002). Some components of the fungal calcium-calcineurin signaling pathway have been demonstrated to be potential and effective targets for the development of new antifungal drugs because these proteins are vital to fungal growth, survival, and drug tolerance (Sanglard et al., 2003; Liu et al., 2015). However, Ca^2+^ influx-activated calcineurin in mammalian cells and *C. albicans* can also induce apoptosis (Wang et al., 1999; Lu et al., 2010), and the inherent calcineurin inhibitor FKBP38 can inhibit apoptosis (Shirane and Nakayama, 2003). In our study, the disruption of calcium homeostasis in carvacrol-treated *C. albicans* cells suggested that Ca^2+^/calcineurin might be activated and involved in the apoptotic process of the carvacrol-treated *C. albicans* cells. To confirm this inference, the calcium-scavenger BAPTA, calcineurin inhibitor CsA, and calcineurin mutant *cmp1*Δ*/*Δ were applied. As expected, BAPTA, CsA and *cmp1*Δ*/*Δ significantly lowered carvacrol-mediated metacaspase activity, indicating that carvacrol induced *C. albicans* apoptosis by Ca^2+^/calcineurin pathway.

Various animal models, including an oral candidiasis model (Chami et al., 2004b), vaginal candidiasis model (Chami et al., 2004a), and murine systemic candidiasis model (Manohar et al., 2001), have been used to validate the efficacy of carvacrol against fungal infection *in vivo*. For example, Chami et al. (2004b) reported a significant CFU decrease in samples collected from the oral cavity of carvacrol-treated rats compared with untreated control rats. Moreover, there was no hyphal colonization of the epithelium in rats treated with carvacrol, indicating that carvacrol might be a strong antifungal agent for treatment of oral candidiasis (Chami et al., 2004b). Similarly, Chami et al. (2004a) also demonstrated that both prophylactic and therapeutic treatment with carvacrol can eradicate the vaginal fungal burden of infected rats, suggesting that carvacrol can be considered a promising compound in the treatment of vaginal candidiasis (Chami et al., 2004a). Manohar et al. (2001) observed that carvacrol treatment significantly increased the survival rate of infected mice in an experimental murine systemic candidasis model. Most of the above researches were conducted with immunosuppressed animals or did not consider immunomodulation activities. However, in our study, we found carvacrol was also able to modulate immunity in addition to kill fungi, which might better explain the antifungal effect of carvacrol in diminishing *C. albicans* infections.

There are some limitations to our present study. The compound we utilized is not particularly novel, considering there have been studies documenting the effects of carvacrol against *C. albicans* infection. However, our study is novel in its elucidation of the mechanisms through which carvacrol induces *C. albicans* cell death. In addition, this study did not conduct a histological analysis, which is a more direct approach to visualizing the inflamed areas and hyphae or yeast in the infected kidneys. Nevertheless, evidence from the current study substantiates the safety and effectiveness of carvacrol as a therapy for treating systemic *C. albicans* infections.

## Data Availability Statement

The raw data supporting the conclusions of this article will be made available by the authors, without undue reservation, to any qualified researcher.

## Ethics Statement

Healthy ICR mice (female, 25–30 g) were supplied by Wenzhou Medical University (License No. SCXK [ZJ] 2005–0019). All procedures that involved animals were conducted in accordance with *The Guide for the Care and Use of Laboratory Animals* of the China National Institutes of Health. These procedures were authorized by the Animal Care and Use Committee of Wenzhou Medical University (wydw 2017-0046). All efforts were made to minimize the suffering of animals used in the present research.

## Author Contributions

CN, CW, and YY participated in the design and execution of most of the experiments, analysis and interpretation of the data, and drafting and revising the manuscript. RC, JZ, HC, and YZ were responsible for evaluating the cell membrane, metacaspase activity, DNA fragmentation, and calcium level assays. JL, JC, KX, and MC assessed mitochondrial function and performed the animal experiments. CR, CZ, and CJ participated in manuscript revision and supervision of the work.

## Conflict of Interest

The authors declare that the research was conducted in the absence of any commercial or financial relationships that could be construed as a potential conflict of interest.

## Abbreviations

CFUs: colony forming Units
CsA: cyclosporine A
DCFH-DA: 2′,7′-dichlorofluorescein diacetate
DiBAC_4_(3): bis-(13-dibarbituric acid)-trimethine oxanol
HBSS: Hank's balanced salt solution
ICR: Institute of Cancer Research
JC-1: 5,5′,6,6′-tetrachloro-1,1′,3,3′-tetraethyl-benzimidazolyl carbocyanine iodide
MIC: minimal inhibitory concentration
OG: oral-gastric
PI: propidium iodide
ROS: reactive oxygen species
TUNEL: Terminal deoxynucleotidyl transferase dUTP nick end labeling
YPD: yeast extract peptone dextrose.
